# Dopamine D_1_, D_2_, D_3_ Receptors, Vesicular Monoamine Transporter Type-2 (VMAT2) and Dopamine Transporter (DAT) Densities in Aged Human Brain

**DOI:** 10.1371/journal.pone.0049483

**Published:** 2012-11-21

**Authors:** Jianjun Sun, Jinbin Xu, Nigel J. Cairns, Joel S. Perlmutter, Robert H. Mach

**Affiliations:** 1 Department of Radiology, Washington University School of Medicine, St. Louis, Missouri, United States of America; 2 Department of Neurology, Washington University School of Medicine, St. Louis, Missouri, United States of America; 3 Department of Pathology and Immunology, Washington University School of Medicine, St. Louis, Missouri, United States of America; 4 Department of Neurobiology, Washington University School of Medicine, St. Louis, Missouri, United States of America; 5 Department of Occupational Therapy, Washington University School of Medicine, St. Louis, Missouri, United States of America; 6 Department of Physical Therapy, Washington University School of Medicine, St. Louis, Missouri, United States of America; 7 Department of Cell Biology amd Physiology, Washington University School of Medicine, St. Louis, Missouri, United States of America; 8 Biochemistry and Molecular Biophysics, Washington University School of Medicine, St. Louis, Missouri, United States of America; Centre for Addiction and Mental Health, Canada

## Abstract

The dopamine D_1_, D_2_, D_3_ receptors, vesicular monoamine transporter type-2 (VMAT2), and dopamine transporter (DAT) densities were measured in 11 aged human brains (aged 77–107.8, mean: 91 years) by quantitative autoradiography. The density of D_1_ receptors, VMAT2, and DAT was measured using [^3^H]SCH23390, [^3^H]dihydrotetrabenazine, and [^3^H]WIN35428, respectively. The density of D_2_ and D_3_ receptors was calculated using the D_3_-preferring radioligand, [^3^H]**WC-10** and the D_2_-preferring radioligand [^3^H]raclopride using a mathematical model developed previously by our group. Dopamine D_1_, D_2_, and D_3_ receptors are extensively distributed throughout striatum; the highest density of D_3_ receptors occurred in the nucleus accumbens (NAc). The density of the DAT is 10–20-fold lower than that of VMAT2 in striatal regions. Dopamine D_3_ receptor density exceeded D_2_ receptor densities in extrastriatal regions, and thalamus contained a high level of D_3_ receptors with negligible D_2_ receptors. The density of dopamine D_1_ linearly correlated with D_3_ receptor density in the thalamus. The density of the DAT was negligible in the extrastriatal regions whereas the VMAT2 was expressed in moderate density. D_3_ receptor and VMAT2 densities were in similar level between the aged human and aged rhesus brain samples, whereas aged human brain samples had lower range of densities of D_1_ and D_2_ receptors and DAT compared with the aged rhesus monkey brain. The differential density of D_3_ and D_2_ receptors in human brain will be useful in the interpretation of PET imaging studies in human subjects with existing radiotracers, and assist in the validation of newer PET radiotracers having a higher selectivity for dopamine D_2_ or D_3_ receptors.

## Introduction

The dopaminergic system is involved in neurological disorders such as Parkinson disease, drug addiction and schizophrenia [1]–[4]. Dopamine receptors have been classified into two subtypes: D_1_-like and D_2_-like receptors. Stimulation of D_1_-like (D_1_ and D_5_) receptors activates adenylate cyclase and increases cAMP (cyclic adenosine monophosphate) production. Stimulation of D_2_-like (D_2_, D_3_ and D_4_) receptors inhibits adenylate cyclase activity, increases arachadonic acid release and phosphatidylinositol hydrolysis [5], [6]. The dopamine transporter (DAT) is a presynaptic membrane protein which is responsible for the reuptake of dopamine into dopaminergic nerve terminals. The vesicular monoamine transporter type-2 (VMAT2) is a vesicular membrane protein that transport monoamines from the cytosol into synaptic vesicles [7]. Both have been used as dopamine presynaptic markers for nigrostriatal neuronal integrity.

Since radioligands for PET imaging dopamine D_2_-like receptors, such as the antagonists [^11^C]raclopride [8], [^18^F]fallypride [9] and the full agonist [^11^C](+)-PHNO [10], bind to both the dopamine D_2_ and D_3_ receptors, PET studies can only measure the composite density of these receptors, the dopamine D_2_/D_3_ receptor binding potential. Quantitative autoradiography measuring dopamine D_2_ and D_3_ receptor densities have yielded equivocal receptor density values and distribution patterns in human and monkey brain [11]–[18]. This can be attributed to the low D_2_/D_3_ selectivity of all radioligands used in these studies. Some studies have attempted to quantify dopamine D_3_ receptors using “selective” radiolabeled dopamine D_3_ agonists (7-OH-DPAT, PIPAT and PD128947), but these ligands also bind to the high affinity agonist binding state of the D_2_ receptor and require first decoupling the D_2_ receptor from G proteins to image the D_3_ receptor. Studies using radiolabeled selective dopamine D_3_ versus D_2_ receptor antagonists are not well documented [5], [18], [19].

**WC-10**, a weak partial agonist/antagonist at the D_3_ receptor, binds with a 66-fold higher affinity to human HEK D_3_ than HEK D_2L_ receptors, with a dissociation constant (*K_d_*) of 1.2 nM at HEK D_3_ receptors [19], [20]. By using [^3^H]**WC-10** and a D_2_/D_3_ ligand [^3^H]raclopride, we have developed a quantitative autoradiography assay for measuring the absolute densities of dopamine D_2_ and D_3_ receptors in the striatal regions of rat and rhesus monkey brain [18]. In this study, the absolute densities of dopamine D_2_ and D_3_ receptors were determined by using the same autoradiography assay in the striatal and extrastriatal regions of an aged monkey (25 years old) and aged human brains (average age = 91, range = 77–107.8 years old). The dopamine D_1_ receptor, DAT, and VMAT2 densities were also measured by quantitative autoradiography. The results of this study provide a unique measurement of the density of D_1_, D_2_ and D_3_ receptors, and DAT and VMAT2 levels, in the same human brain samples.

## Materials and Methods

### Ethics Statement

After death, the written consent of the next of kin was obtained for brain removal, following local Ethical Committee procedures (Human Studies Committee, Washington University School of Medicine). Postmortem receptor autoradiography study has been approved by the Alzheimer's disease Research Center (ADRC) Committee; the approval letter is submitted as a supplement.

The monkey used in this study belongs to our group and was euthanized using pentobarbital 100 mg/kg i.v. due to age-related health decline. This method is consistent with the recommendations of the Panel on Euthanasia of the American Veterinary Medical Association. These studies have been approved by the IACUC at Washington University (approval #20110161). Washington University is fully accredited by the American Association for the Accreditation of Laboratory Animal Care (AAALAC).

### Precursor synthesis and radiolabeling

**Figure 1:**
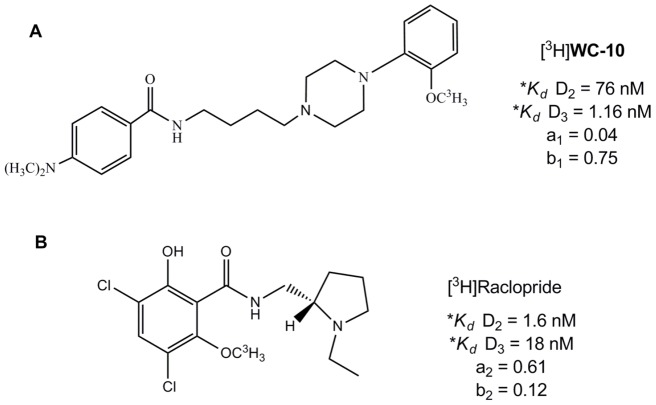
Chemical structures of [^3^H]WC-10 and [^3^H]raclopride. *K_d_* values were obtained through saturation binding of [^3^H]**WC-10** and [^3^H]raclopride to cloned human D_3_ and D_2L_ receptors expressed in HEK cells. a_1_, and b_1_ represent the fractional receptor occupancy to dopamine D_2_ and D_3_ receptors in human brain at a ligand concentration of 3.54 nM for [^3^H]**WC-10**. a_2_ and b_2_ represent the same parameters at a ligand concentration of 2.50 nM [^3^H]raclopride. The receptor occupancy fractions were calculated from the saturation binding isotherm using the *K_d_* values. *Data were taken from Xu et al. (2009).

[^3^H]**WC-10** (Figure 1) was synthesized by American Radiolabeled Chemicals (St Louis, Missouri, USA) by alkylation of the desmethyl precursor with [^3^H]methyl iodide. The specific activity of the radioligand was 80 Ci/mmol. The detailed synthesis scheme for [^3^H]**WC-10** has been previously described [19].

### Drugs

Chemical reagents and the standard compounds were purchased from Sigma (St. Louis, MO) and Tocris (Ellisville, MO). [^3^H]raclopride (76 Ci/mmol), [^3^H]SCH23390 (85 Ci/mmol) and [^3^H]WIN35428 (76 Ci/mmol) were purchased from Perkin Elmer Life Sciences (Boston, MA). [^3^H]dihydrotetrabenazine ([^3^H]DTBZ) (20 Ci/mmol) was purchased from American Radiolabeled Chemicals (St Louis, Missouri, USA).

### Tissue collection

Clinically and neuropathologically well-characterized human brain tissues were obtained from the Knight Alzheimer's Disease Research Center, Washington University School of Medicine. All cases were longitudinally assessed, healthy elderly individuals without neurological or psychiatric disease and included 4 males and 7 females, aged 77–107.8 (mean: 91) years. Table 1 shows the demographic case variables. Brains were removed at autopsy and the right hemibrain was coronally sliced and snap-frozen by contact with Teflon-coated aluminum plates cooled in liquid nitrogen vapor, subsequently stored in zip-lock airtight plastic bags and stored at −80°C until used. Microscopy was performed using established rating scales. Alzheimer's disease pathological changes were assessed using Braak staging [21], [22]. For autoradiography studies, frozen coronal sections (20 µm) were cut with a Microm cryotome and mounted on Superfrost Plus glass slides (Fisher Scientific, Pittsburgh, PA) from following brain regions: precommissural striatal regions containing the caudate, putamen and nucleus accumbens (NAc); globus pallidus (GP) containing the internal and external part (GPi and GPe); thalamus containing postcommissural striatal regions; and middle brain containing substantia nigra (SN) and red nucleus (RN). For the determination of total binding, data from 2–4 sections were averaged and nonspecific binding was defined by average of 1–2 adjacent sections for all the radioligands. Another set of adjacent sections used for cresyl violet staining to identify related anatomical structures.

**Table 1:** Demographic details of human brains.

Autopsy number	Age(y)	Gender	Brain Weight(g)	PMI(h)	Braak NFT	Braak Amyloid	CDR
105.460	77	F	1410	10	1	A	0
107.050	96	M	1165	12	1	0	0
100.060	91.6	F	1310	16	2	0	0
105.210	107.8	F	1080	5	2	A	0
101.200	92.1	F	1120	6	0	0	0
104.300	91.6	F	1220	16	2	A	0
11.027	79	F	1100	25	1	A	0
11.041	100	F	1450	21	2	C	0
10.740	90	M	1150	10	4	A	0
10.150	84	M	1010	5.5	1	B	0
9.255	91	M	1170	8.5	1	A	0

PMI: Post-mortem interval; CDR: Clinical dementia rating.

### Quantitative autoradiography protocol

Sections for dopamine D_1_, D_2_, and D_3_ receptor binding were pre incubated for 20 min at room temperature in buffer (50 mM Tris buffer, pH 7.4, containing 120 mM NaCl, 5 mM KCl) to remove endogenous dopamine. After incubation with the respective radiotracer, slides were then rinsed five times at 1 min intervals with ice-cold buffer. Slides were incubated in an open staining jar, with the free radioligand concentration loss at less than 5% as previously described [18], [19].

#### Dopamine D_1_ receptor binding

D_1_ receptors were labeled with [^3^H]SCH23390 using the procedure described by Savasta [23] with minor modifications. Briefly, after preincubation to remove endogenous dopamine, sections were incubated for 60 min at room temperature in a similar buffer solution with the addition of 1.44 nM [^3^H]SCH23390 and 30 nM ketanserin tartrate (Tocris Bioscience, Ellisville, Missouri, USA) to block 5-HT_2_ receptors. Nonspecific binding was determined in the presence of 1 µM (+)-butaclamol as described previously [24], [25].

#### Dopamine D_2_ receptor binding

D_2_ receptors were labeled with [^3^H]raclopride using the previously described procedure for rat and monkey tissue [18]. Brain sections were incubated for 60 min in buffer solution at room temperature with the addition of 2.50 nM [^3^H]raclopride. Nonspecific binding was determined from the slides in the presence of 1 µM *S*-(–)-eticlopride [18].

#### Dopamine D_3_ receptor binding

D_3_ receptors were labeled with [^3^H]**WC-10** using the previously described procedure for rat and monkey tissue [18]. Brain sections were incubated for 60 min in buffer solution at room temperature with the addition of 3.54 nM [^3^H]**WC-10**, 10 nM WAY-100635 was added to solution to block 5-HT_1A_ receptors. Nonspecific binding was determined in the presence of 1 µM *S*-(–)-eticlopride [18].

#### DAT binding

DAT were labeled with [^3^H]WIN35428. Brain sections were incubated for 60 min in buffer solution at room temperature with the addition of 2.19 nM [^3^H]WIN35428. Nonspecific binding was determined from the slides in the presence of 1 µM nomifensine.

#### VMAT2 binding

VMAT2 binding sites were labeled with [^3^H]DTBZ. Brain sections were incubated for 60 min in buffer solution at room temperature with the addition of 4.53 nM [^3^H]DTBZ. Nonspecific binding was determined from the slides in the presence of 1 µM *S*-(–)tetrabenazine.

#### Quantification of total radioactivity

Slides were air dried and made conductive by coating the free side with a copper foil tape. Slides were then placed into a gas chamber containing a mixture of argon and triethylamine (Sigma-Aldrich, USA) as part of a gaseous detector system, the Beta Imager 2000Z Digital Beta Imaging System (Biospace, France). After the gas was well mixed and a homogenous state was reached, further exposure for 20 h yielded high-quality images. A [^3^H]Microscale (American Radiolabeled Chemicals, St Louis, Missouri, USA) was counted simultaneously as a reference for total radioactivity quantitative analysis. Quantitative analysis was performed with the program Beta-Vision Plus (BioSpace, France) for each anatomical region of interest.

#### Cresyl violet staining

A set of adjacent sections was fixed with 4% paraformaldehyde for 10 min, washed with PBS for 1 min, then dipped in 100% ethanol for 20 seconds to remove fat and fixation chemicals. Sections were then stained with 0.5% cresyl violet solution for 3 min, washed in running tap water 10 min, dehydrated by a series of alcohol baths, and made transparent by xylene (2×4 min) and scanned with an Epson scanner.

#### Determination of absolute densities of D_2_ and D_3_ receptors

Measurement of the absolute densities of dopamine D_2_ and D_3_ receptors using the D_3_ selective radioligand [^3^H]**WC-10** and the D_2_/D_3_ ligand, [^3^H]raclopride was described previously [18]. Briefly, the receptor fractional occupancy of [^3^H]**WC-10** and [^3^H]raclopride to human dopamine D_2_ and D_3_ receptors can be calculated by the saturation binding isotherm:
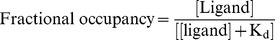
The total amount of receptor bound for [^3^H]**WC-10** and [^3^H]raclopride can be expressed by formula:
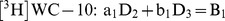


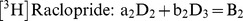
Where a_1_ and b_1_ are the fractional occupancies of [^3^H]**WC-10** to D_2_ and D_3_ receptors; B_1_ is the total receptor density (D_2_/D_3_) directly measured from autoradiography studies of [^3^H]**WC-10**; a_2_, b_2_, and B_2_ are the same parameters for [^3^H]raclopride; D_2_, D_3_ is the absolute density of D_2_ and D_3_ receptors. The absolute densities of D_2_ and D_3_ receptors can be calculated by solving the simultaneous equations:
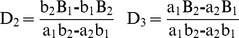



#### Statistical analysis

The receptor-bound radioligand binding apparent densities were calculated using the specific activity of each radioligand expressed as fmol/mg tissue as previously described [18]. The experimenter was blinded to all conditions during the analysis. Comparison of receptor densities was analyzed by an unpaired Student's t test. Assessment of correlation between different receptors binding was calculated using Pearson product moment correlation coefficient.

## Results

### Quantitative autoradiography

The sensitivity limit of Beta Imager 2000Z Digital Beta Imaging System is 0.07 dpm/mm^2^. A tritium standard [^3^H]Microscale with a known amount of radioactivity (ranging from 0 to 36.3 nCi/mg) was counted with each section and used to create a standard curve; in each case the standard curve had a correlation coefficient (R) greater than 0.99. On the basis of the saturation binding analysis and the *in vitro* binding data of [^3^H]**WC-10** and [^3^H]raclopride to cloned human D_2_ and D_3_ receptors [19], *K_d_* value and fractions of D_2_ and D_3_ receptor occupancies with 3.54 nM [^3^H]**WC-10** and 2.50 nM [^3^H]raclopride binding in human brain can be readily determined. The values of *K_d_* and receptors occupancies fractions are summarized in Figure 1.

### Quantitative analysis of dopamine D_1_, D_2_, D_3_ receptors, DAT and VMAT2 densities in aged human brain

**Figure 2:**
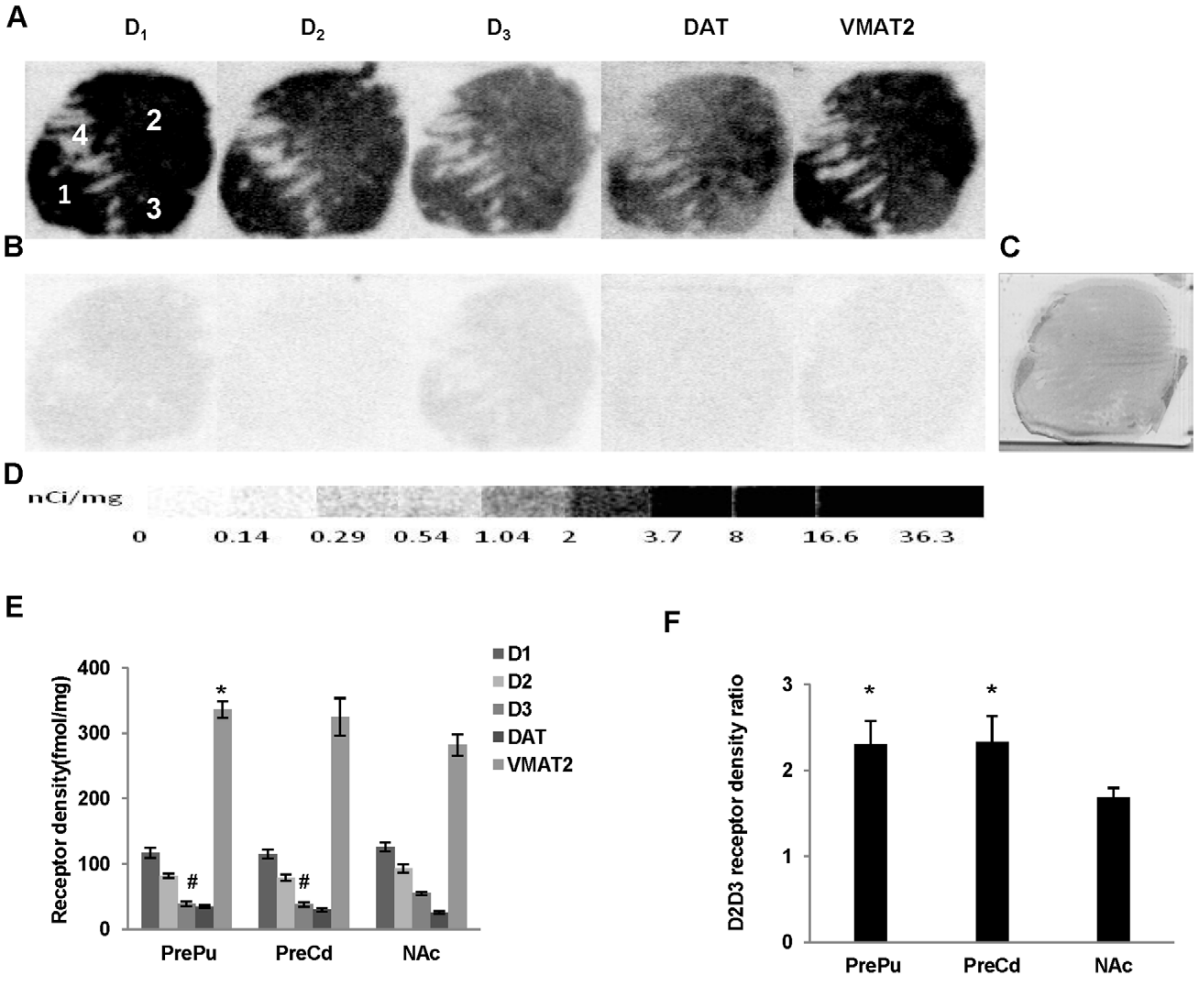
Quantitative autoradiographic analysis of dopamine receptors, DAT and DTBZ densities in the precommissural striatal regions. Autoradiograms show total binding of 1.44 nM [^3^H]SCH23390, 2.50 nM[^3^H]raclopride, 3.54 nM [^3^H]**WC-10**, 2.19 nM [^3^H]WIN35428, and 4.53 nM [^3^H]DTBZ (A), and nonspecific binding in presence of 1 µM (+) butaclamol (for [^3^H]SCH23390), 1 µM *S*(-)-eticlopride (for [^3^H]raclopride and [^3^H]**WC-10**), 1 µM nomifensine (for WIN35428) and 1 µM *S*(-)-tetrabenazine (for DTBZ) (B) in the precommissural striatal regions of human brain sections. The adjacent section shows cresyl violet staining to identify related anatomical structures (C). [^3^H]Microscale standards (ranging from 0 to 36.3 nCi/mg) were also counted (D). Quantitative analysis of dopamine D_1_, D_2_, and D_3_ receptors, and DAT and DTBZ densities (fmol/mg) and the dopamine D_2_ ∶ D_3_ receptor density ratio in human striatal regions are shown in E and F respectively. The numbers 1 through 4 designate the following CNS anatomical regions: 1: Precommissural Putamen (PrePu); 2: Precommissural caudate (PreCd);3: Nucleus accumbens (NAc); 4: Internal capsule (IC). *p<0.05, #p<0.01 compared to NAc.

**Figure 3:**
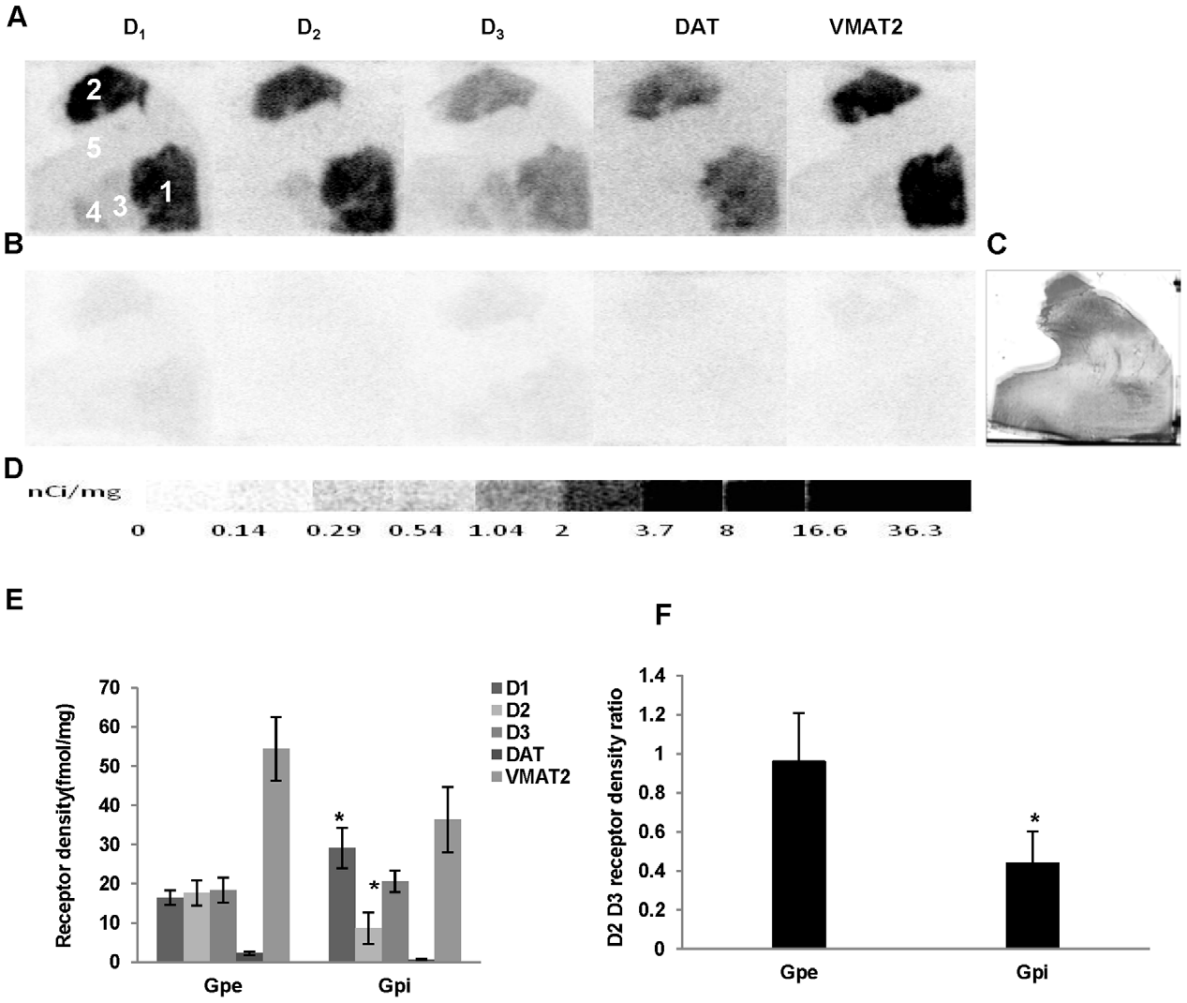
Quantitative autoradiographic analysis of dopamine D_3_ receptors, DAT and DTBZ densities in the globus pallidus. Autoradiograms show total binding of 1.44 nM [^3^H]SCH23390, 2.50 nM [^3^H]raclopride, 3.54 nM [^3^H]**WC-10**, 2.19 nM [^3^H]WIN35428, 4.53 nM [^3^H]DTBZ (A), and nonspecific binding in presence of 1 µM (+) butaclamol (for [^3^H]SCH23390), 1 µM *S*(-)-eticlopride (for [^3^H]raclopride and [^3^H]**WC-10**), 1 µM nomifensine (for [^3^H]WIN35428) and 1 µM *S*(-)-tetrabenazine (for [^3^H]DTBZ) (B) in the globus pallidus of aged human brain sections. The adjacent section shows cresyl violet staining to identify related anatomical structures (C). [^3^H]Microscale standards (ranging from 0 to 36.3 nCi/mg) were also counted (D). Quantitative analysis of dopamine D_1_, D_2_, and D_3_ receptors, DAT and DTBZ densities (fmol/mg) and the dopamine D_2_ ∶ D_3_ receptor density ratio in human globus pallidus are shown in E and F, respectively. The numbers 1 through 5 designate the following CNS anatomical regions: 1: Putamen; 2: Caudate; 3: Globus pallidus external part (GPe); 4: Globus pallidus internal part (GPi); 5: Internal capsule (IC). *p<0.05 compared to GPe.

**Figure 4:**
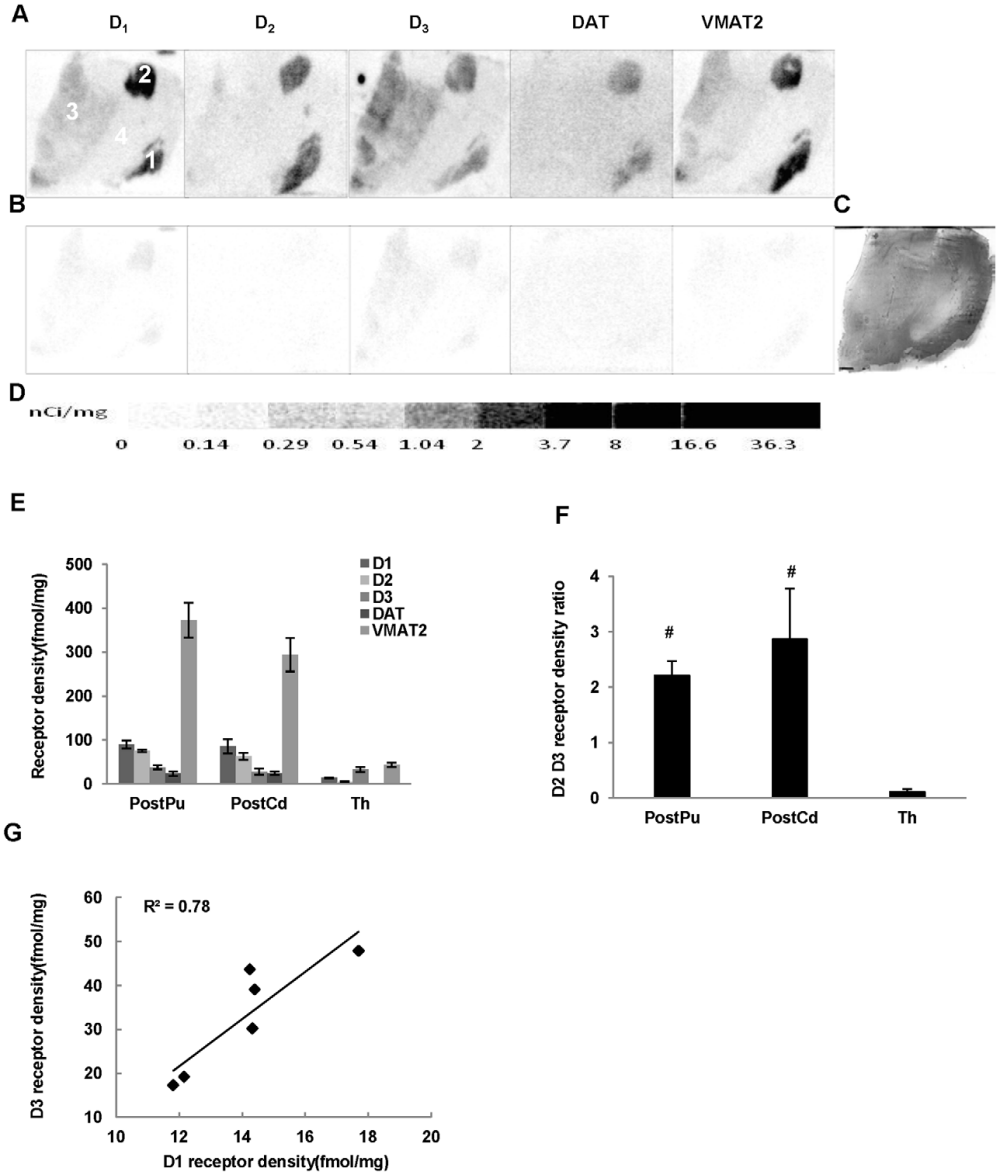
Quantitative autoradiographic analysis of dopamine receptors, DAT and DTBZ densities in the thalamus. Autoradiograms show total binding of 1.44 nM [^3^H]SCH23390, 2.50 nM [^3^H]raclopride, 3.54 nM [^3^H]**WC-10**, 2.19 nM [^3^H]WIN35428, 4.53 nM [^3^H]DTBZ (A), and nonspecific binding in presence of 1 µM (+) butaclamol (for [^3^H]SCH23390), 1 µM *S*(-)-eticlopride (for [^3^H]raclopride and [^3^H]**WC-10**), 1 µM nomifensine (for WIN35428) and 1 µM *S*(-)-tetrabenazine (for DTBZ) (B) in the thalamus of human brain sections. The adjacent section shows cresyl violet staining to identify related anatomical structures (C). [^3^H]Microscale standards (ranging from 0 to 36.3 nCi/mg) were also counted (D). Quantitative analysis of dopamine D_1_, D_2_, and D_3_ receptors, DAT and DTBZ densities (fmol/mg) and the dopamine D_2_ ∶ D_3_ receptor density ratio in human brain are shown in E and F, respectively. Linear correlation analysis of the average dopamine D_1_ and D_3_ receptor densities in human thalamus is shown in (G). The numbers 1 through 4 designate the following CNS anatomical regions: 1: Postcommissural putamen (PostPu); 2: Postcommissural caudate (PosCd); 3: Thalamus; 4: Internal capsule (IC). #p<0.01 compared to thalamus.

**Figure 5:**
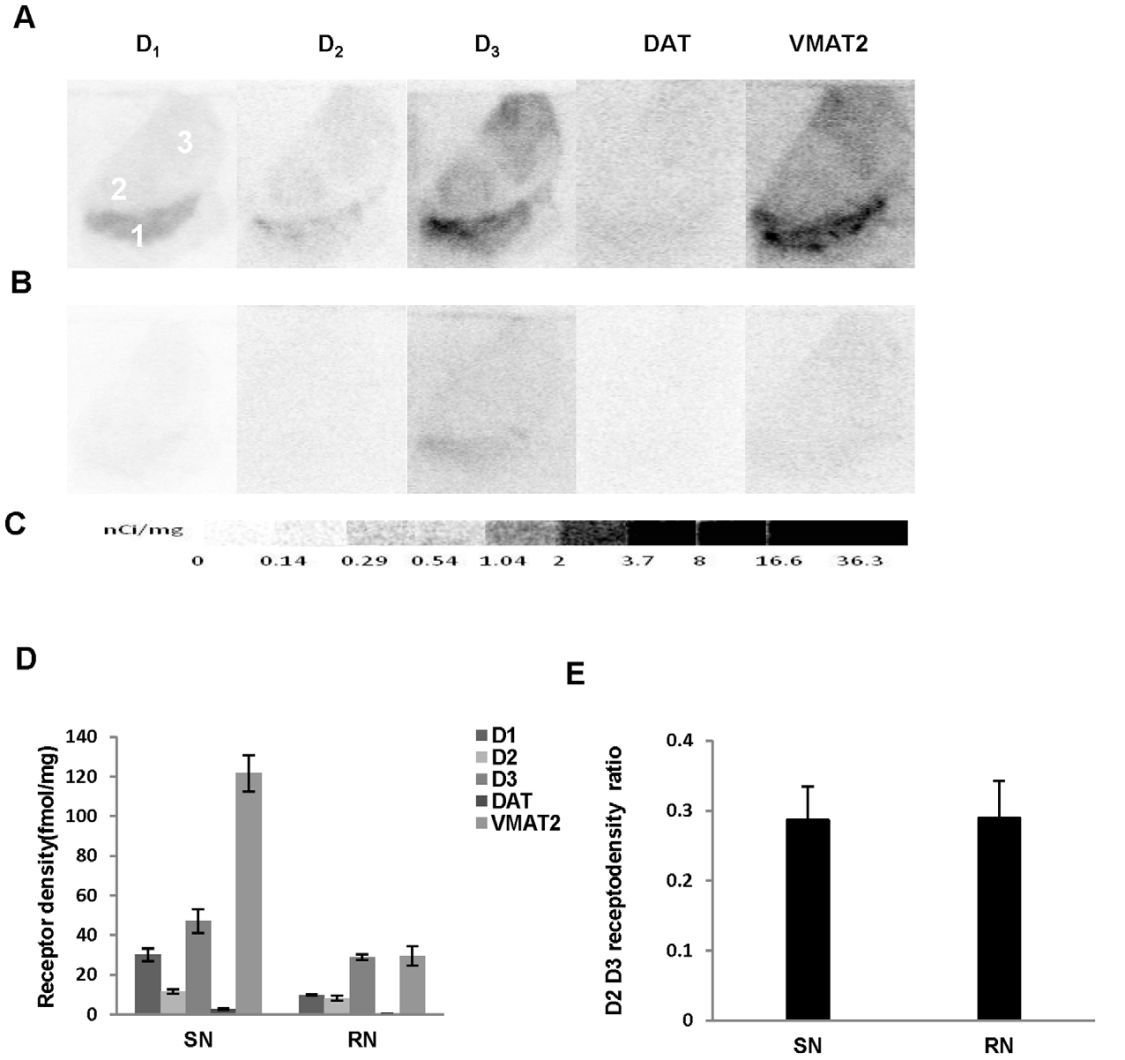
Quantitative autoradiographic analysis of dopamine receptors, and DAT and DTBZ densities in the substantia nigra. Autoradiograms show total binding of 1.44 nM [^3^H]SCH23390, 2.50 nM[^3^H]raclopride, 3.54 nM [^3^H]**WC-10**, 2.19 nM [^3^H]WIN35428, 4.53 nM [^3^H]DTBZ (A), and nonspecific binding in presence of 1 uM (+) butaclamol (for [^3^H]SCH23390), 1 µM *S*(-)-eticlopride (for [^3^H]raclopride and [^3^H]**WC-10**), 1 µM nomifensine (for WIN35428) and 1 µM *S*(-)-tetrabenazine (for DTBZ) (B) in the substantia nigra (SN) of aged human brain sections. [^3^H]Microscale standards (ranging from 0 to 36.3 nCi/mg) were also counted (C). Quantitative analysis of dopamine D_1_, D_2_ and D_3_ receptors, and DAT and DTBZ densities (fmol/mg) and the dopamine D_2_ ∶ D_3_ receptor density ratio in human SN and red nucleus are shown in D and E respectively. The numbers 1 through 3 designate the following CNS anatomical regions: 1: Substantia nigra (SN); 2: Red nucleus (RN); 3: Thalamus.

The binding densities of dopamine D_1_ receptor, DAT and VMAT2 were determined by quantitative autoradiography using 1.44 nM [^3^H]SCH23390, 2.19 nM [^3^H]WIN355428 and 4.53nM [^3^H]DTBZ, respectively. The apparent receptor binding densities (B_1_ and B_2_) of D_2_ plus D_3_ receptors were measured by using 3.54 nM [^3^H]**WC-10** and 2.50 nM [^3^H]raclopride respectively, and the absolute D_2_ and D_3_ receptors densities were determined as described above. The nonspecific binding was determined by using different high affinity cold compounds (Figures 2B, 3B, 4B, 5B). The receptor density values are summarized in Table 2.

**Table 2:** Dopamine D_1_, D_2_, D_3_ receptors, dopamine transporter (DAT) and vesicular monoamine transporter type-2 (VMAT2) densities and D_2_ ∶ D_3_ receptor density ratio in aged human brain.

Region	Region abbreviation	DAT	VMAT2	D_1_	D_2_	D_3_	D_2_D_3_ ratio	n
Precommissural putamen	PrePu	35±3	336±13	117±7	82±3	39±3	2.30±0.28	10
Precommissural caudate	PreCd	30±4	325±29	115±7	79±5	38±4	2.29±0.27	10
Nucleus accumbens	NAc	26±5	282±16	126±7	93±6	55±2	1.69±0.11	8
Postcommissural putamen	PosPu	24±5	373±39	90±9	76±3	38±5	2.21±0.27	7
Postcommissural caudate	PosCd	24±3	294±38	86±16	63±7	28±7	2.87±0.91	4
Globus pallidus external part	GPe	2±0.4	54±8	16±2	18±3	18±3	0.96±0.25	6
Globus pallidus internal part	GPi	0.6±0.3	36±8	29±5	9±4	21±3	0.44±0.16	6
Thalamus	Th	0.8±0.4	44±5	14±1	4±2	33±5	0.11±0.05	6
Substantia nigra	SN	4±0.4	122±9	30±3	13±1	47±6	0.30±0.05	8
Red nucleus	RN	4±0.3	30±5	10±0.9	8±1	29±1	0.29±0.05	6

Receptor densities (fmol/mg) presented as mean value ±SEM.

#### Precommissural striatal regions

Dopamine D_1_, D_2_ and D_3_ receptors were found to be extensively distributed throughout the precommissural striatal regions. The dopamine D_3_ receptor density was much lower than that of the D_1_ and D_2_ receptors (Table 2; Figure 2). The dopamine D_3_ receptor density was significantly lower in the putamen (p = 0.001) and caudate (p = 0.0001) than that of the NAc (Figure 1E). No difference in the D_3_ receptor density was found between the putamen and caudate. The dopamine D_2_∶D_3_ receptor density ratio was significantly higher in the putamen (p = 0.04) and caudate (p = 0.04) compared to that of the NAc, but was not different between the caudate and putamen (Figure 1F). The VMAT2 density was found to be ∼10-fold higher than that of DAT in this region. Densities of DAT and VMAT2 were similar among the three sections of the precommissural striatal regions; an exception was the putamen, which showed significant increase in VMAT2 density versus that of the NAc (P = 0.01) (Figure 1E).

#### Globus pallidus

The density of dopamine D_1_ and D_2_ receptors, and DAT and VMAT2 were dramatically lower in the GP, whereas the density of the dopamine D_3_ receptor was just slightly lower when compared to those of the striatal regions (Table 2; Figure 3). The distribution of dopamine receptors was different between the GPe and GPi: the dopamine D_1_, D_2_, and D_3_ receptor densities were similar in the GPe, while the dopamine D_1_ receptor density was significantly higher (p = 0.02) and the D_2_ receptor density was significantly lower (p = 0.03) in the GPi compared to that of GPe (Figure 3E). Because of the lower density of dopamine D_2_ receptors in the GPi, the dopamine D_2_∶D_3_ receptor density ratio was significantly lower than that of GPe (Figure 3F). A lower level of VMAT2 density was distributed in both regions of the GP, whereas the density of the DAT was negligible compared to that in the striatal regions (Table 2; Figure 3E).

#### Thalamus

Dopamine D_1_ receptor density was much lower and the D_2_ receptor was negligible in the thalamus compared to those of the striatal regions (Table 2; Figure 4A, E). In contrast, the dopamine D_3_ receptor density exceeded that observed in striatal regions, resulting in a low D_2_∶D_3_ receptor density ratio in the thalamus (0.11±0.05) compared to that of the striatal regions (Figure 4F). A strong linear correlation (R^2^>0.78) between the average density of dopamine D_1_ and D_3_ receptors was found in the thalamus (Figure 4G). A lower level of VMAT2 was found in the thalamus, whereas DAT density was nearly zero (Table 2; Figure 4A, E).

#### Postcommissural striatal regions

There were no significant differences in dopamine D_2_ and D_3_ receptor densities, and the D_2_∶D_3_ receptor density ratio, between the pre- and postcommissural striatal regions. However, the dopamine D_1_ receptor density was found to be significantly lower in the postcommissural putamen (p = 0.01) and caudate (p = 0.01) compared to their precommissural counterparts. The DAT density was found to be significantly decreased in the post- versus precommissural putamen (p = 0.04), while the VMAT level did not change.

#### Substantia nigra

Dopamine D_1_ and D_2_ receptor densities were much lower in the SN compared to those of the striatal regions. In contrast, the dopamine D_3_ receptor density in the SN was the highest among the extrastriatal regions, and is only slightly lower than that of NAc (Table 2; Figure 5D). Consequently, the dopamine D_2_∶D_3_ receptor density ratio in the SN was very low (Figure 5E). There was a moderate density of VMAT2 in the SN, while DAT density was negligible in this region (Figure 5D).

#### Red nucleus

Receptor densities in red nucleus (RN) were extremely low except for the dopamine D_3_ receptor, which showed a relatively high density in this area (Table 2; Figure 5D). The dopamine D_2_∶D_3_ receptor density ratio in the RN was similar as that of SN (Figure 5E).

### Comparison of dopamine D_1_, D_2_, and D_3_ receptors, and DAT and VMAT2 densities in the striatal regions between aged rhesus monkey and aged human brain

**Figure 6:**
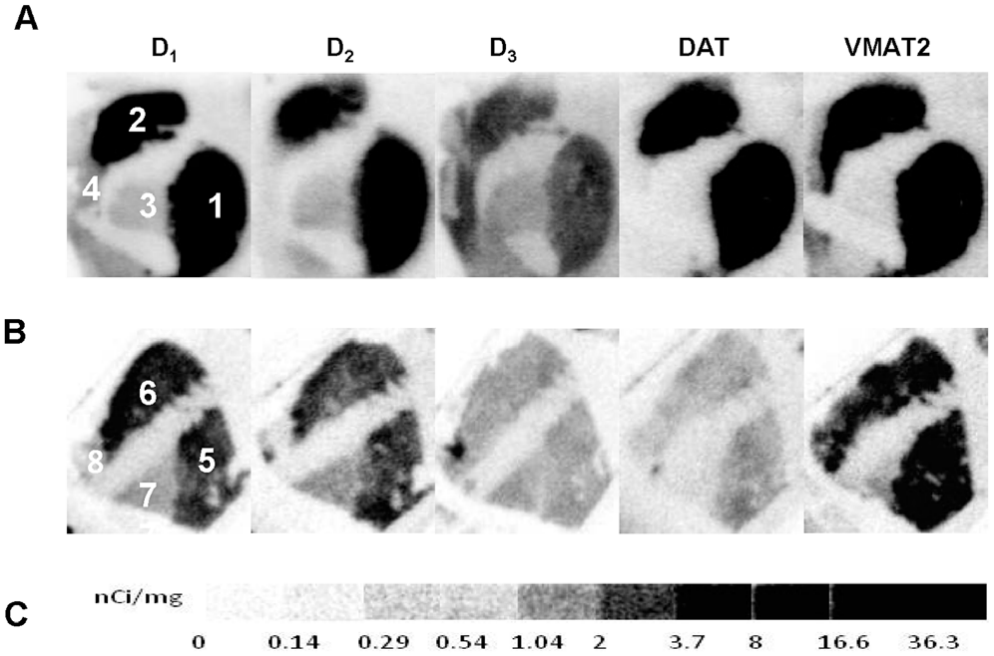
Comparison of dopamine D_1_, D_2_, and D_3_ receptors, and DAT and DTBZ densities in the striatal regions between an aged rhesus monkey (25 years old) and aged human brain samples. Autoradiograms show neuroanatomical localization of [^3^H]SCH23390 for D_1_, [^3^H]raclopride for D_2_, [^3^H]**WC-10** for D_3_ receptors, [^3^H]WIN35428 for DAT and [^3^H]DTBZ for VMAT2 in the striatal regions of rhesus monkey (A) and aged human brain (B). [^3^H]Microscale stnadards (ranging from 0 to 36.3 nCi/mg) (C). The numbers 1 through 8 in panels (A) (B) designate the following CNS anatomical regions: 1: Monkey putamen; 2: Monkey caudate; 3: Monkey globus pallidus; 4: Monkey thalamus; 5: Human putamen; 6: Human caudate; 7: Human globus pallidus; 8: Human thalamus.

To investigate the species differences of dopamine receptors and presynaptic markers, we compared the density of dopamine D_1_, D_2_, and D_3_ receptors and DAT and VMAT2 in the striatal regions of an aged rhesus monkey (25 years old ) to those of aged human brain (average age: 91 years old). The densities of dopamine D_1_ and D_2_ receptors and DAT were found to be lower in aged human brain compared to those of rhesus monkey, whereas the dopamine D_3_ receptor and VMAT2 densities were similar between these two species (Table 3; Figure 6).

**Table 3:** Comparison of dopamine D_1_, D_2_, D_3_ receptors, DAT and VMAT2 densities (fmol/mg) in the striatal regions of adult rhesus monkey and aged human brain.

	D_1_		D_2_		D_3_		DAT		VMAT2	
	Pu	Cd	Pu	Cd	Pu	Cd	Pu	Cd	Pu	Cd
Rhesus monkey	256±19	228±9	178±9	205±6	36±9	46±4	185±12	177±17	341±20	351±10
Human	117±23	119±16	82±11	77±15	39±11	36±11	35±10	30±11	336±40	325±86

Data were obtained from 10 aged healthy human and a 25 years old rhesus monkey brain and presented as mean value ± stdev. Pu: Putamen; Cd: Caudate.

### Different regulation of VMAT2 and DAT in the striatal regions and substantia nigra of aged human brain

**Figure 7:**
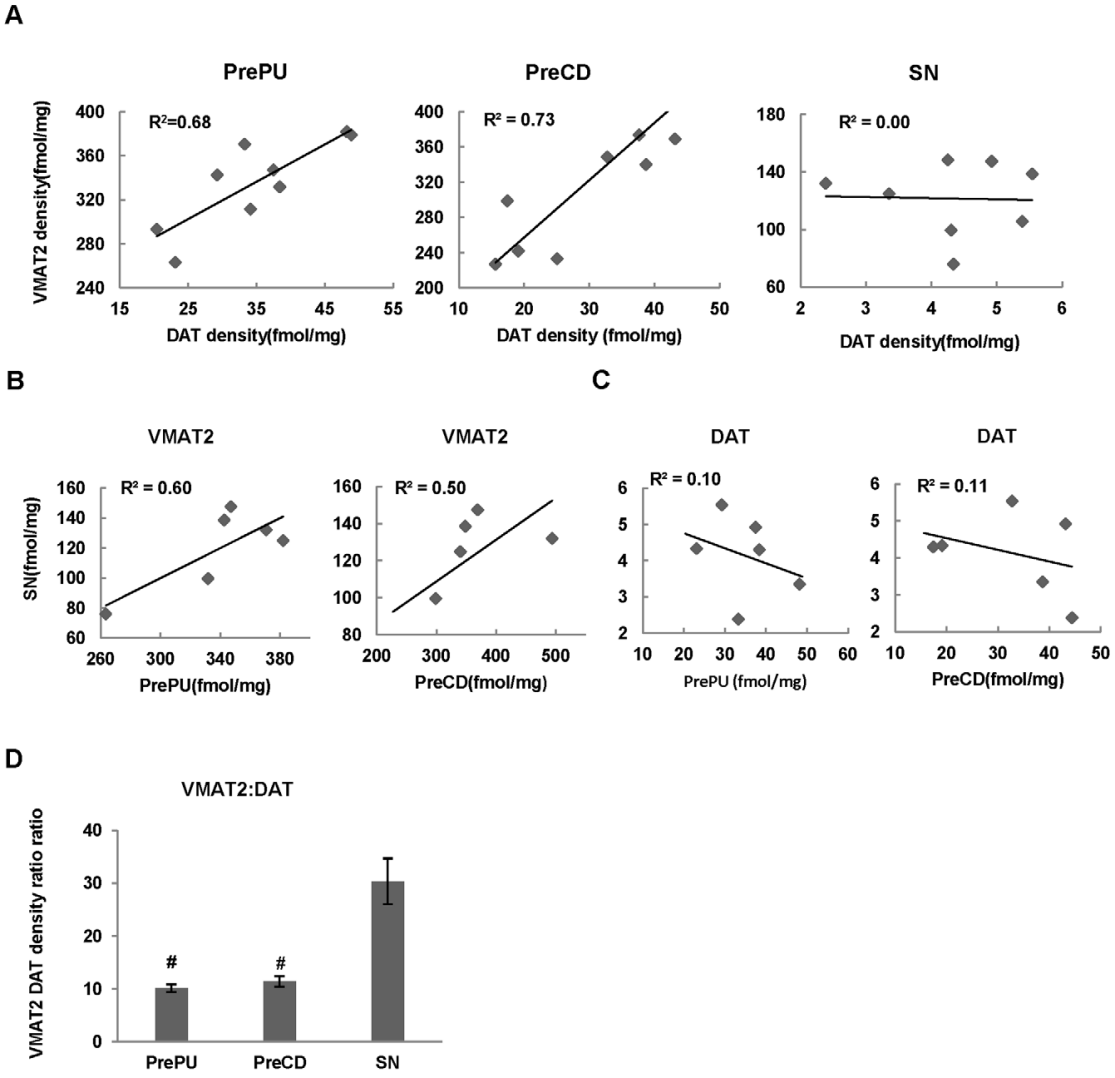
Correlation of DAT with VMAT2 in the striatal regions and substantia nigra. The correlation between the VMAT2 and DAT densities in the precommissural putamen (PrePu), caudate (PreCd) and substantia nigra (SN) (A). Correlation of the VMAT densities between the substantia nigra (SN) and PrePu or PreCd (B). Correlation of the DAT densities between the SN and PrePu or PreCd (C). The average VMAT DAT density ratio in the PrePu, PreCd and SN (D). #p<0.01 compared to SN.

In all brain regions measured, the VMAT2 density was found to be significantly higher than that of the DAT (Table 2). The VMAT2∶DAT density ratio was regionally-dependent: the VMAT2 density was 30-fold higher than that of the DAT in the SN but only 10-fold higher in the precommissural striatal regions (Figure 7D). The average VMAT2 density strongly linearly correlated with DAT densities in the precommissural putamen (r^2^ = 0.68) and caudate (r^2^ = 0.73), but not in the SN (r^2^<0.01) (Figure 7A). The VMAT2 density in the SN significantly correlated with those in the precommissural putamen (r^2^ = 0.60) (Figure 7B) and caudate (r^2^ = 0.50), but no such correlation was found for the DAT either in the precommissural putamen (r^2^ = 0.10) or caudate (r^2^ = 0.11) (Figure 7C).

## Discussion

Our group had previously reported the density of dopamine D_2_ and D_3_ receptors in rat and rhesus monkey brain using a novel autoradiography method involving the use of two different radioligands, the D_3_-preferring ligand [^3^H]WC-10 and the D_2_/D_3_ nonselective ligand [^3^H]raclopride [18]. Here we report first measurements of D_2_ and D_3_ specific receptors in aged human postmortem brain. We also included measurements of the density of dopamine D_1_ receptors, DAT and VMAT2 using well-established tritiated ligands and quantitative autoradiography. Some noteworthy findings include: 1) D_3_ receptors were widely distributed throughout the striatal and extrastriatal regions in the aged human brain; 2) in the striatal regions, D_3_ receptors were more enriched in the NAc than in the caudate and putamen; 3) in the extrastriatal regions, dopamine D_3_ receptor density exceeded D_2_ receptors; 3) DAT density in aged human brain was more than 10-fold lower than that of VMAT2 in the striatal regions, and was negligible in the SN, whereas VMAT2 density was relatively high; 4) receptor densities of dopamine D_1_, D_2_ and DAT was lower in human versus monkey brain, but D_3_ and VMAT2 densities appeared to be similar.

Quantitative autoradiography to measure dopamine D_3_ receptor density have previously been conducted using radiolabeled agonists such as [^3^H]7-OH-DPAT, [^125^I]PIPAT, and [^3^H]PD 128907. Since these ligands bind to both the D_3_ receptor and the dopamine “high affinity binding site” of the D_2_ receptor [26], the D_2_ receptor must first be “decoupled” to form the dopamine low affinity agonist binding state in order to measure D_3_ receptors with these radioligands [13]–[15], [27], [28]. Other studies have used radiolabeled D_2_/D_3_ antagonists in the presence of a D_2_-preferring antagonist to determine the density of D_2_ and D_3_ receptors in autoradiography studies [12], [16], [17]. However, it is difficult to quantify D_2_ and D_3_ receptor density using this approach given the relatively low D_2_/D_3_ selectivity of the D_2_-preferring blocking agent. Recently our lab has developed a new radiolabeled D_3_ receptor antagonist/partial agonist [^3^H]WC-10, which has high binding affinity and selectivity to D_3_ versus D_2_ receptors [19], [20]. By combining autoradiography studies with [^3^H]WC-10 with the D_2_/D_3_ nonselective ligand [^3^H]raclopride, the density of dopamine D_2_ and D_3_ receptors can be easily determined using the mathematical model [18].

The current finding of the dopamine D_3_ receptor distribution pattern in the striatal regions is in agreement with some previous reports [13], [14], [18], but not consistent with other reports demonstrating a restricted distribution in the limbic areas of the striatum [17], [29], [30]. However, in situ hybridization studies have shown that dopamine D_3_ receptor mRNA is found in the caudate, putamen and nucleus accumbens in human and monkey brain [13], [31], [32], which provides additional support for the current observations. The distribution of dopamine D_3_ receptors in the putamen and caudate, with a higher density in the NAc, suggests that the dopamine D_3_ receptor may also be involved in the regulation of locomotor function in addition to their well-recognized role in the limbic system.

The measurement of dopamine D_3_ receptors in the GPi is consistent with previous publications [17]. Interestingly, the dopamine D_1_ receptor density was found to be significantly higher and the D_2_ receptor density significantly lower in the GPi versus GPe, which is in agreement with the recent finding showing the similar distribution of dopamine D_1_ and D_2_ receptors in the globus pallidus by using bacterial artificial chromosome (BAC) transgenic mice in which expression of enhanced green fluorescent protein (eGFP), is driven by the promoter region of either the D_1_ or the D_2_ [33]. The different distribution pattern of the dopamine D_1_ and D_2_ receptors in the GPe and GPi found in this study has provided the additional proof that the D_1_ receptormediated direct pathway going from striatum to GPi and the D_2_ receptor mediated indirect pathway going from striatal to GPe.

The thalamus is another interesting target for brain dopamine [34]. Previous receptor autoradiography studies with the radioligand [^125^I]epidepride found a modest density of dopamine D_2_-like receptors in the thalamus [12], [35]. On the other hand, dopamine D_3_ receptor density was found to be very low in human thalamus when [^3^H]7-OH-DPAT was used as the radioligand [13]. In PET imaging studies, radiotracers such as [^18^F]fallypride which has a high affinity for both D_2_ and D_3_ receptors, and [^11^C]PHNO which is a D_3_ preferring ligand, , display a high uptake in the thalamus of human and monkey brain [9], [36]–[40]. This is in contrast to [^18^F]NMB and [^11^C]raclopride, both tracers have lower uptakes in the thalamus versus the striatum and putamen [41], [42]; NMB and raclopride have higher affinities for D_2_ versus D_3_ receptors, which could explain their relatively low uptake in the thalamus. In the present study, dopamine D_3_ receptors were found to be abundant while the D_2_ receptor was nearly negligible in the thalamus of aged human brain. Consequently, the dopamine D_2_∶D_3_ receptor density ratio was very low in this area. Although the current autoradiography study was conducted in aged subjects with no sign of neurological disease, it is not likely that the aging process would result in a complete loss of dopamine D_2_ receptors in lieu of D_3_ receptors. Therefore, our data indicate that the thalamus can be used as a good region to study dopamine D_3_ receptor function in PET imaging studies using radiotracers such as [^18^F]fallypride, [^11^C]raclopride, and [^11^C]PHNO. It should be noted that differences in the D_2_/D_3_ binding potential of these PET radiotracers in the thalamus have been reported in a variety of neurological and neuropsychiatric disorders, including schizophrenia [43]–[46], substance abuse [47], [48] and dystonia [49], [50] relative to age-matched controls. The finding of the high D_3_ receptor density and low D_2_∶D_3_ ratio in the human thalamus indicates that the changes of D_2_/D_3_ thalamic binding potential in these patients measured by PET may be attributed to changes in dopamine D_3_ receptor function, and that dopamine D_3_ receptors may play a key role in the pathophysiology of these disorders.

The dopamine D_3_ receptor was also found to be abundantly distributed in the SN and RN, whereas the density of D_2_ receptors was lower. Dopamine D_2_-like receptors were observed in the RN and SN with high and moderate density in a human PET imaging study using [^11^C]FLB 457 [51]. More recent studies using the dopamine D_3_-preferring agonist [^11^C]PHNO [38], reported a high density of dopamine D_3_ receptors and negligible D_2_ receptors in the SN [40], [52]–[55], which is consistent with our autoradiography findings. The dopamine D_1_ receptor was also found to be present in the SN with a density intermediate to D_3_ and D_2_ receptors, which agrees with previous reports [56]–[58]. The abundance of dopamine D_3_ receptors and lower D_2_∶D_3_ receptor density ratio in human SN represents a second region to study dopamine D_3_ versus D_2_ effects using currently available PET ligands. The functional significance of the abundant existence of D_3_ versus D_2_ receptors in human SN is not clear. One possible explanation is that dopamine D_3_ receptors may be involved in the negative feedback regulation of tonic dopamine release.

A number of biochemical and behavioral studies have suggested that D_1_ and D_3_ receptors may functionally interact [59], [60]. For example, D_1_ and D_3_ mRNAs are co-localized in a large number of neurons in the striatum [61] and the NAc [62]–[64], and co-activation of D_1_ and D_3_ receptors in the shell of the NAc synergistically increases substance P expression [63], [64]. D_1_ and D_3_ interactions are thought to mediate the rewarding properties of low doses of cocaine [36], and L-DOPA administration to rats receiving a unilateral injection of the neurotoxin 6-OH-dopamine results in an overexpression of D_3_ receptors in nigrostriatal neurons that constitutively express D_1_ receptors [59], [65], [66]. Dopamine D_1_ and D_3_ receptors were co-expressed in the renal proximal tubule [67] and in transfected HEK-293 cells [68]. Heterodimerization of these two receptors has been observed by co-immunoprecipitation from striatal protein preparations [59] or by bioluminescence resonance energy transfer technique in transfected mammalian cells [59], [68]. It is of interest to note that a linear correlation of dopamine D_1_ and D_3_ receptor densities was found in the thalamus, which is consistent with either an anatomical or functional coupling of D_1_ and D_3_ dopamine receptor subtypes. D_1_ receptors are not thought to interact with D_3_ receptors functioning as autoreceptors [60]; and we found no strong correlation of D_1_ and D_3_ receptor density in the caudate, putamen and SN (data not shown), where D_3_ receptors may function as autoreceptors. The availability of [^3^H]**WC-10**, a D_3_-preferring radioligand, will provide a valuable tool for studying the functional interactions between dopamine D_1_ and D_3_ receptors in the CNS.

The DAT and VMAT2 distribution pattern found in this study is consistent with the previous reports [69]–[72].The higher density level of both DAT and VMAT2 was found in the putamen and caudate compared to that of nucleus accumbens, which is in line with the recent finding using the same radioligands [73]. Surprisingly the DAT density was 10 fold lower than that of VMAT2 in aged human striatum which is different from previous reports. Furthermore the DAT density was lower while the VMAT2 density was not significantly different in the aged human compared to that of monkey brain. This may reflect the different aging related change patterns of these two dopamine presynaptic markers. In fact aging related decline of DAT but not VMAT2 density in the human brain has been reported [74]–[76]. In the striatal regions, the DAT density was significantly correlated with that of the VMAT2, indicating the anatomical and functional coupling of these two presynaptic dopamine markers.

The monkey brain had a higher density of D_1_ and D_2_ receptors relative to the human brain, but a similar density of D_3_ receptors. Dopamine D_1_ [58], [77]–[79] and D_2_ [46], [80]–[82] receptor densities decline with aging in human brain, but no reports have been published measuring the density of D_3_ receptors as a function of age.

The main limitation of this study is absence of data from younger subjects. The results of this study may only reflect the densities and distribution of the dopamine receptors and transporters in advanced aged human brain, and may not reflect age-related changes of presynaptic and postsynaptic dopamine markers. Therefore, caution should be given when comparing these data with that of PET imaging studies of the dopaminergic in younger subjects.

## Conclusions

This study provides quantitative measurements of the density of presynaptic (VMAT2 and DAT) and postsynaptic dopaminergic markers (dopamine D_1_, D_2_, and D_3_ receptors) in the aged human brain. The correlation between the density of D_1_ and D_3_ receptors in the thalamus, and the DAT and VMAT2 in the striatal regions, suggests a functional interaction between these markers. The high density of D_3_ receptors in the thalamus and SN and low density of D_2_ receptors in these brain regions could provide valuable information for PET imaging D_3_ and D_2_ receptor function using [^18^F]fallypride, [^11^C]raclopride, [^18^F]NMB, and [^11^C]PHNO. The differential density of D_2_ and D_3_ receptors in these brain regions can also be used in determining the *in vivo* selectivity on newer PET radiotracers which are being developed to discriminate between D_3_ and D_2_ receptors and vice versa.
